# A dynamical pattern recognition model of gamma activity in auditory cortex

**DOI:** 10.1016/j.neunet.2011.12.007

**Published:** 2012-04

**Authors:** M. Zavaglia, R.T. Canolty, T.M. Schofield, A.P. Leff, M. Ursino, R.T. Knight, W.D. Penny

**Affiliations:** a Wellcome Trust Centre for Neuroimaging, University College, London WC1N 3BG, UK; b Department of Electronics, Computer Science and Systems (DEIS), Via Venezia 52, 47023 Cesena, Italy; c Helen Wills Neuroscience Institute, University of California, Berkeley, CA, USA; d Institute of Neurology and Institute of Cognitive Neuroscience, UCL, 17 Queen Square, London WC1N 3AR, UK

**Keywords:** Gamma activity, Speech recognition, Synchronization, Transients, Coupled-oscillator, Bayesian estimation

## Abstract

This paper describes a dynamical process which serves both as a model of temporal pattern recognition in the brain and as a forward model of neuroimaging data. This process is considered at two separate levels of analysis: the algorithmic and implementation levels. At an algorithmic level, recognition is based on the use of Occurrence Time features. Using a speech digit database we show that for noisy recognition environments, these features rival standard cepstral coefficient features. At an implementation level, the model is defined using a Weakly Coupled Oscillator (WCO) framework and uses a transient synchronization mechanism to signal a recognition event. In a second set of experiments, we use the strength of the synchronization event to predict the high gamma (75–150 Hz) activity produced by the brain in response to word versus non-word stimuli. Quantitative model fits allow us to make inferences about parameters governing pattern recognition dynamics in the brain.

## 1 Introduction

Hopfield and Brody (2000, 2001) (HB) have proposed a model for how the brain might recognize spatiotemporal patterns, and have applied it to the problem of auditory word recognition. Their model is particularly appealing at two different levels of analysis (Marr & Poggio, 1976).

First, at an ‘algorithmic’ level the HB model uses a preprocessing stage comprising a bank of filters and a set of feature detectors which signal onsets, offsets and peak activities in different frequency ranges. This is broadly consistent with the physiology of the mammalian auditory system (Casseday, Fremouw, & Covey, 2002; Ghitza, 1986). The key aspect of their algorithm, however, is that the subsequent pattern recognition is based on the Occurrence Times (OTs) of features which provides a natural invariance to the speed at which a word is spoken.

Second, at an ‘implementation’ level the recognition of OTs is achieved using a transient synchronization mechanism. This phenomenon relies on a combination of three physiological processes acting in concert (i) spike rate adaptation, (ii) synaptic plasticity and (iii) neuronal synchronization. In the HB model synchronization arises via balanced excitation and inhibition (Tsodyks, Mitkov, & Sompolinsky, 1993) in a network of Integrate and Fire (IF) cells. Together these mechanisms provide a burst of gamma activity that corresponds to a ‘recognition event’. This is particularly interesting to imaging neuroscientists as bursts of gamma activity (which we define here to be higher than 30 Hz in frequency) have been observed to accompany auditory word recognition (Canolty et al., 2007; Lutzenberger, Pulvermuller, & Birbaumer, 1994; Pulvermuller et al., 1996).

This paper draws heavily on the HB model and makes three new contributions to the literature. First, we consider the algorithmic level and use a speech database to assess the usefulness of OT features as compared to standard features used in Automatic Speech Recognition (ASR) that are based on cepstral coefficients (Rabiner & Juang, 1993). Both types of features (OT or cepstral) are then used as input to an identical pattern recognition module. This allows us to assess the usefulness of the features themselves independently of the utility of the pattern recognition process or its putative neurobiological implementation.

Second, we propose a more generic model of transient synchronization based on a Weakly Coupled Oscillator (WCO) framework (Hoppensteadt & Izhikevich, 1997). WCOs are a standard approach for studying synchronization dynamics (Hoppensteadt & Izhikevich, 1997) and can be derived by applying a phase reduction approach to neurophysiologically realistic neural (Gutkin, Ermentrout, & Reyes, 2005) or neural network (Brown, Moehlis, & Holmes, 2004) models. The only requirement is that the underlying neurons operate around a limit cycle and interact weakly (Brown et al., 2004; Ermentrout & Kleinfeld, 2001; Hansel, Mato, & Meunier, 1995).

This paper uses a WCO model of transient synchronization which we refer to as the WCO–TS model. As in the HB model, recognition is signalled by a transient synchronization event, and this synchronization is brought about by coupling feature detectors that have nonstationary, pattern-dependent frequency response profiles. However, the synchronization process itself is not implemented using balanced excitation and inhibition among IF cells as in Hopfield and Brody (2001), but is rather described at the level of phase dynamics. This allows us to be equivocal about the details of the neural circuits that generate the oscillations themselves. We see this as a benefit as there are currently a large number of possible candidates for the underlying processes (see next section).

Third, we show how the WCO–TS model can be directly fitted to neuroimaging data. This follows the example of ‘Dynamic Causal Modelling’ in which differential equation models of physiological processes are fitted to data and scored against each other using Bayesian inference (Friston, Harrison, & Penny, 2003; Girolami, 2008; Penny, Litvak, Fuentemilla, Duzel, & Friston, 2009; Penny, Stephan, Mechelli, & Friston, 2004). Specifically, we show how the WCO–TS model can be used as a forward model of gamma activity observed in Electrocorticographic (ECOG) data.

The paper is organized as follows. The following subsection briefly reviews the topics of gamma activity and network synchronization. Section 2.1 then describes the ECOG data and the spectral analysis methods used to find the underlying gamma burst associated with word recognition. This is based on previous work (Canolty et al., 2007). Section 2.2.6 then describes the WCO–TS model and how it is fitted to data. The results section reports on the efficacy of OT features as assessed using a spoken digit database, and on the use of WCO–TS as a forward model of ECOG data.

### 1.1 Gamma activity and synchronization

The phenomenon of gamma activity has received tremendous interest in imaging neuroscience. It initially rose to prominence with regard to the feature binding problem, whereby features of the same object that are represented in different brain regions must somehow be tied together to form a coherent whole. It was proposed that synchronization between the relevant regions at gamma frequency was just such a mechanism (Singer, 1999). There has since been a large amount of work in this area with reviews focusing on its role in large-scale integration (Varela, Lachaux, Rodriguez, & Martinerie, 2001), enhanced communication (Fries, 2005), attention and memory (Jensen, Kaiser, & Lachaux, 2007) and spike-timing dependent plasticity (Buzsaki, 2006). Gamma is also the single frequency band which most strongly predicts BOLD activity (Goense & Logothetis, 2008). We are therefore interested in gamma activity as it potentially provides a connection between computational and imaging neuroscience.

In the auditory domain several studies have found stronger (25–35 Hz) gamma responses to words as opposed to pseudo-words (Lutzenberger et al., 1994; Pulvermuller et al., 1996) and in the 60–70 Hz range to words as opposed to non-words (Eulitz et al., 1996). Additionally, Canolty et al. (2007) have found High Gamma (80–200 Hz) responses in ECOG recordings to words as opposed to non-words. Additionally, this High Gamma activity occurred sequentially over posterior Superior Temporal Gyrus (STG), mid STG, followed by Superior Temporal Sulcus (STS). This extends previous findings from fMRI (Binder et al., 2000) and provides evidence for a degree of seriality in word processing. It is this data set that we will analyse using the WCO–TS model.

The above neuroimaging results and related conceptual advances have motivated a number of theoretical models. For example, Shamir, Ghitza, Epstein, and Kopell (2009) have developed a neurophysiologically realistic model that shows how gamma oscillations can directly represent stimuli whose time scale is longer than a single gamma cycle, as is required for the representation of auditory words. Hopfield (2004) shows that subthreshold oscillations can be used to support a spike-time based code that leads to minimal interference with coexisting firing rate codes, and that subthreshold oscillations at gamma frequency may be important for encoding of speech. This principle has been developed by Ghitza (2007) who also propose that hierarchies of rhythms may be the mechanism by which the brain integrates information over multiple time scales during language processing.

We now turn to the issue of what is the physiological origin of gamma activity. As with most oscillatory phenomena in the brain, gamma is thought to arise from a combination of factors (i) a cell’s intrinsic ability to oscillate, (ii) the presence of feedback connections among groups of excitatory and inhibitory neurons and (iii) the ability of networks of cells to either amplify or nullify certain oscillations. These factors are described in a recent comprehensive review (Wang, 2010). One mechanism for network amplification is the synchronization of cell activity.

The frequency of oscillations produced by single cells is determined primarily by the synaptic time constants and levels of driving input, with faster synapses and stronger inputs generally leading to higher frequency oscillations. These oscillations require that cells receive a tonic excitatory drive. When two cells are connected the resulting activity depends on whether the intervening interactions are fast or slow.

Mathematical studies of coupled oscillators show that for fast interactions, synchronization is most readily achieved using excitatory connections (Vreeswijk, Abbott, & Ermentrout, 1994). In the mammalian brain fast excitatory connections can be mediated by electrical synapses or gap junctions. These are found, for example, between pyramidal cells in hippocampus. In neural network models with tonic drive, gap junctions can lead to synchronized gamma activity (Pfeuty, Mato, Golomb, & Hansel, 2003). Traub, Schmitz, Jefferys, and Draguhn (1999), have shown using simulations that a network of pyramidal cells, electrically coupled through their axons, can generate High Gamma activity without chemical synapses.

If the interactions are slow then synchronization is most readily achieved using inhibitory connections. Chemical synapses with realistic rise times fall into this ‘slow’ category. For a pair of IF cells receiving tonic excitation, synchronization can be achieved using mutual inhibition (Vreeswijk et al., 1994). This result follows over to conductance-based models with large numbers of cells (Tiesinga & Jose, 2000; Wang & Buzsaki, 1996; White, Chow, Ritt, Soto-Trevino, & Kopell, 1998). These network models are referred to as Inhibitory Network Gamma (ING) oscillators (Bartos, Vida, & Jonas, 2007). ING oscillators have slow synapses and connections are weak. For these oscillations to impact on signals sent from a region they must recruit pyramidal cells which then in turn re-excite local interneurons. This results in so-called Pyramidal Inhibitory Network Gamma (PING) oscillators (Whittington, Traub, Kopell, Ermentrout, & Buhl, 2000).

A potential problem with ING/PING oscillators is that they are sensitive to parameter inhomogeneities between cells. If cells receive different input drives then synchronization can be destroyed (Wang & Buzsaki, 1996). Gamma oscillations that are resistant to such inhomogeneities, however, can be generated with ING oscillators having strong rather than weak synapses, fast rather than slow synapses, and with inhibition that is shunting (i.e., vetoing any excitatory input) rather than merely hyperpolarizing (Bartos et al., 2007). In mammalian neocortex the fastest synapses exist in the form of gap junctions between layer 4 inhibitory interneurons. These junctions promote synchronization without changing network frequency (Bartos et al., 2007).

In the networks we have so far described both excitatory and inhibitory cells fire, approximately, on every gamma cycle so that the Local Field Potential (LFP) oscillation frequency is the same as the firing rate of the cells. Brunel and Hakim (1999) have investigated a different regime they call Weak Stochastic Synchronization (WSS) in which interneurons and pyramidal cells fire stochastically and during a small proportion of gamma cycles only. WSS can be brought about by combining strong synapses with noise. This work has been extended to models with more realistic synaptic kinetics (Brunel & Wang, 2003) and conductance-based models (Geisler, Brunel, & Wang, 2005).

There is also a body of work showing that stochastically driven Neural Mass Models (David & Friston, 2003) comprising stellate cells, interneurons and pyramidal cells, can generate a range of frequencies including gamma. These have recently been extended by incorporating an additional population of reciprocally connected fast interneurons (Ursino, Cona, & Zavaglia, 2010). This results in a robust model of gamma activity using realistic synaptic time constants.

For more detailed mechanisms underlying gamma oscillations we refer the reader to recent reviews (Bartos et al., 2007; Buzsaki, 2006; Wang, 2010; Whittington et al., 2000). The point here is that there is a diversity of possible network mechanisms (PING/ING, gap junctions, WSS) underlying gamma activity. We also emphasize that gamma is not a unitary phenomenon and probably has different underlying mechanisms depending on, among other factors, experimental context, computational role, anatomical location etc. Importantly, almost all of the above work has focused on sustained gamma oscillations, whereas the current paper focuses on transient gamma activity. One key distinction is that the inhomogeneities in input drive that reduce the robustness of a sustained gamma oscillation are not a problem for transient dynamics. Indeed this very feature can be made use of to signal a pattern recognition event—only when the input drives are similar will synchronization occur. This endows the Hopfield and Brody model and the WCO–TS network with the specificity necessary for pattern recognition over psychophysiological time scales.

## 2 Materials and methods

### 2.1 Electrocorticogram data

This section describes a spectral analysis applied to the ECOG data presented in Canolty et al. (2007). ECOG data was recorded from an 8-by-8 grid of electrodes placed over fronto-temporal cortex. We analyse data from a single subject, a 37 year old right handed woman with medically intractable complex partial seizures, and from a single electrode (number 49) placed over Superior Temporal Sulcus (STS).

The subject listened to three types of stimuli (i) mouth- or hand-related action verbs, (ii) acoustically matched but unintelligible nonwords and (iii) proper names which served as target stimuli. The subject was instructed to press a button using their left index finger each time they heard a proper name, but not for other stimuli.

The auditory data files (‘.wav’ files) were adjusted to have the same power and duration. Each nonword matched one of the words (action verbs) in duration, intensity and power spectrum, but was rendered unintelligible by removing components of the modulation power spectrum using the Modulation Transfer Function (MTF) algorithm described in Elliott and Theunissen (2009). This is based on a two-dimensional Fourier transform of the log spectrogram, after which slower time–frequency modulations are removed. This results in spectrograms which are, for example, less smooth in the time and frequency domain, as shown for example in the third row of Fig. 3. Further details of the MTF processing are given in Canolty et al. (2007). Overall, our database comprised 96 speech utterances (‘.wav’ files) and 96 matched nonwords.

The ECOG signals were analog filtered between 0.01 and 250 Hz, digitized at a sample rate of 2003 Hz, and high-pass filtered above 2.3 Hz to minimize heartbeat artefact. The data were then epoched from 200 ms before to 1000 ms after stimulus onset to produce i=1–96 time series for words, $y_{wi}$, and for nonwords, $y_{ni}$. Each time series corresponds to processed ECOG data from a single electrode in response to a speech utterance or matched nonword.

The corresponding spectrograms $G\left(y_{wi},f,t\right)$ and $G\left(y_{ni},f,t\right)$ were then computed using a windowed multitaper method with window size N=256 samples (0.128 s), a window offset of 32 samples (0.016 s), and time-bandwidth parameter set to NW=3 (Mitra & Pesaran, 1999). The time-bandwidth parameter is the product of the number of samples N and the frequency resolution, W (in radians). Thus, NW=3 produces a frequency resolution of 3.7 Hz. Each spectrogram was then log-transformed so that power changes over a wide range of frequencies would be visible on the same plot.

**Fig. 1:**
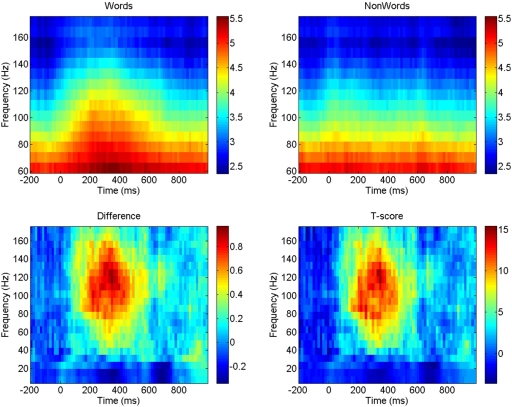
(Top left) Average spectral response to words, (top right) average spectral response to nonwords, (bottom left) difference in spectral response: words–nonwords, (bottom right) significance of difference as assessed with a two-sample t-test. These spectrograms were computed using the multitaper method described in Section 2.1.

Fig. 1 shows the average spectrograms for words and nonwords, the average difference between them (words minus nonwords), and the significance of the difference as assessed using a two-sample t-test. These spectrograms were computed using the multitaper method described above. The figure clearly shows a burst of high-gamma activity (75–150 Hz) for words but not for nonwords. This is exactly the sort of activity that the WCO–TS model predicts should accompany recognition events (see later). The differential spectrogram

(1) $$Y\left(f,t\right)=\frac{1}{N}\overset{N}{\underset{i=1}{\sum }}\left[G\left(y_{wi},f,t\right)-G\left(y_{ni},f,t\right)\right]$$

shown in the bottom left of Fig. 1 is the data feature we wish to explain with the WCO–TS model.

### 2.2 Dynamic pattern recognition model

**Fig. 2:**
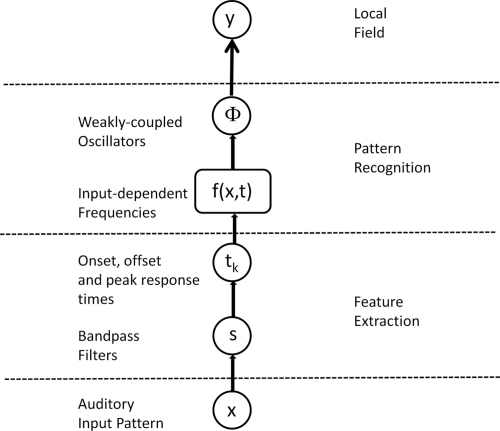
The figure shows the overall processing stream for the dynamic pattern recognition model. This comprises feature extraction, pattern recognition and forward modelling of neuroimaging data. The weakly coupled oscillator dynamics are implemented using Eq. (3). These are driven by stimulus-dependent input frequencies described using Eqs. (2) and (7).

**Fig. 3:**
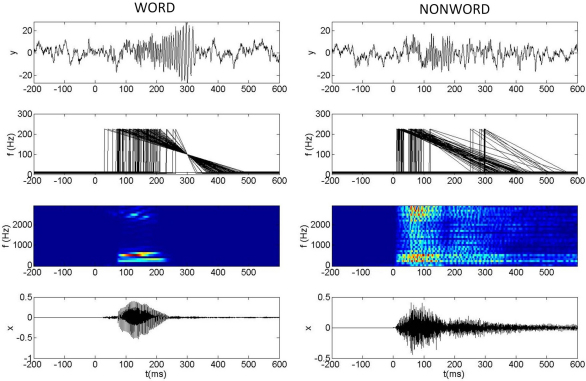
The figure shows auditory inputs x(t) (bottom row), auditory spectrograms s(t) (third row), input frequencies to the WCO–TS network $f_{k}\left(x,t\right)$ (second row), and LFP signals from the WCO–TS net (top row) for a word (left column) and nonword (right column). The times at which the frequencies ramp up to their maximal value (in the second row) are the Occurrence Times (OTs). The word is ‘Hiss’ and the nonword was produced using MTF filtering (see text). The adaptation constants $\tau_{j}$ have been optimized such that the input frequencies become equal at $t_{b}=300\;\mathrm{ms}$ for the word input. The same $\tau_{j}$’s are used to generate the input frequencies for the nonword. For the nonword input there is no time point at which all the frequencies are equal and consequently no large LFP gamma burst.

The overall processing stream for the dynamic pattern recognition model is shown in Fig. 2 and the following subsections describe each step in the processing stream. Briefly, the steps are as follows. First, as described in Section 2.2.1, the original auditory time series (bottom row of Fig. 3) are bandpass filtered into a number of different frequency bands (3rd row of Fig. 3). Second, Occurrence Time (OT) features are extracted as described in Section 2.2.2. These correspond to the times at which power in the different bands cross specified intensity levels. Third, as described in Section 2.2.3, these OT features stimulate activity in a network of feature detectors. Each detector oscillates initially at some maximum frequency which then decreases due to spike rate adaptation, as illustrated in the second row of Fig. 3. Fourth, as described in Section 2.2.4, synaptic plasticity is assumed to have connected together word-specific ensembles that have the appropriate decay constant for each feature such that, at some point post-stimulus, the relevant features are oscillating at the same frequency. This is the ‘Many-Are-Equal (MAE)’ coding scheme proposed by Hopfield and Brody, and the MAE point can be seen in the left column, second row of Fig. 3. Fifth, as described in Section 2.2.5, similar firing rates cause a synchronization event in a network of Weakly Coupled Oscillators which generate a gamma burst in the LFP. This is seen for a recognized auditory word in the left column, top row of Fig. 3 but not for a nonword (right column, top row). Fig. 3 demonstrates the same concept as Fig. 2 in Hopfield and Brody (2001) but uses a WCO rather than IF network. Overall, our dynamical pattern recognition model is identical to the HB model except that the synchronization mechanism is instantiated in a WCO rather than an IF network.

#### 2.2.1 Bandpass filters

Our model assumes that neural circuits in a feature detection region are tuned to specific frequency ranges in a manner that is broadly similar to processing in the cochlear and basilar membrane of the mammalian auditory system. We characterize this activity with b=1..B frequency bins between $f_{\min}$ and $f_{\max}\;\mathrm{Hz}$ where $\omega_{b}$ is the centre frequency of the bth bin. Power in each is computed by bandpassing the input time series, x(t), and then computing the Hilbert envelope $s_{b}\left(t\right)$. Bandpass filters were implemented using Finite Impulse Response (FIR) filters of order 80. Filter coefficients were computed using a least squares criterion and the filters were applied in forward and reverse directions to obtain zero-phase distortion. The filtering was implemented using the firls.m and filtfilt.m functions from the Matlab signal processing toolbox. We emphasize here that these filters are applied to the auditory stimuli rather than to the ECOG data. Example bandpass filter responses to auditory input are shown in the third row of Fig. 3. More physiologically realistic filters can be implemented by linear spacing the filter bands on a mel-frequency scale (Ghitza, 1986), and this was implemented for the pattern recognition results described in Section 3.1.

#### 2.2.2 Occurrence Times

Neurons or neural circuits then respond to three types of features within each frequency band: onsets, offsets and peaks of activity. Such frequency tuned onset and offset detectors have been observed in the inferior colliculus of the auditory midbrain (Casseday et al., 2002). Onset and offset times are computed from the first and last crossings of $s_{b}\left(t\right)$ with a fixed threshold. The peak time is computed from the maximum value of $s_{b}\left(t\right)$. Overall, in response to input pattern x we have K features which are detected at times $t_{k}\left(x\right)$ with k=1..K. For the analysis of the ECOG data (see below) we only use those features for which $t_{k}$ is less than 300 ms, as recognition is required before the end of the word.

Greater physiological realism can be added by using multiple level crossings to define multiple onset and offset points, as in (Ghitza, 1986; Gutig & Sompolinsky, 2009). This adds the property of intensity coding of the auditory signal and was implemented for the pattern recognition results described in Section 3.1. A similar encoding scheme has been proposed by Loiselle, Rouat, Pressnitzer, and Thorpe (2005).

#### 2.2.3 Spike rate adaptation

When a feature is detected the relevant neuron or neural circuit responds by firing at a high frequency, $f_{\max}$, which then decreases. At the single cell level this is known as Spike Rate Adaptation (SRA) or Spike Frequency Adaptation (SFA). Timescales of decay range from tens of milliseconds in the auditory nerve (Zhang, Miller, Robinson, Abbas, & Hu, 2007) to several seconds for delay activity in frontal cortex. Optical imaging reveals larger time windows of temporal integration as one moves from primary to secondary auditory areas (Harrison, Harel, Panesar, Mori, & Mount, 2000). In primary auditory cortex, Ulanovsky, Las, Farkas, and Nelken (2004) have observed within-trial adaptation time constants, at a fast 10 ms time scale, and a slower 150 ms scale. It is these longer time constants that are hypothesized to be useful for auditory object recognition (May & Tiitinen, 2007).

Following HB, for each feature detector we envisage j=1..J neurons or neural circuits each with a different decay constant, $\tau_{j}$. For the jth circuit detecting the kth feature we assume these frequencies are given by linear decays

(2) $$f_{kj}\left(x,t\right)=f_{\max}\left(1-\tau_{j}\left[t-t_{k}\left(x\right)\right]\right)h\left[t-t_{k}\left(x\right)\right]$$

where h[a] is the threshold function with h[a]=1 for a≥0 and zero otherwise. The maximum firing frequency is $f_{\max}$. We have also experimented with exponential decay functions but found that linear functions produced gamma bursts that are better localized in time (and better match ECOG data—see Section 3.2). Overall, the feature detection region comprises C=K×J feature detectors.

#### 2.2.4 Synaptic plasticity

If the input pattern, x, is a word we assume that synaptic plasticity will have acted so as to connect K out of C oscillators together with uniform coupling strength $w_{kk^{\prime }}=A$. This ensemblewill be specialized for recognizing a particular word. Different words will then activate different ensembles of size K in auditory cortex. This is broadly consistent with electrophysiological recordings from non-human primates where representations are composed of small dynamic subsets of highly active neurons (Hromadka, Deweese, & Zador, 2008).

**Fig. 4:**
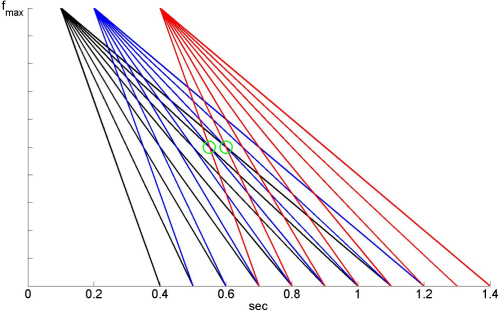
The figure shows the detection of three different input features, k=1,2,3 coloured black, red and blue. The kth feature is detected at time $t_{k}$—these are the Occurrence Times (OTs). For each feature there are j=1..J cells or circuits that initially respond at frequency $f_{\max}$, and then with linearly reducing frequency. The slopes of the frequency reduction are specified by the constants $\tau_{j}$. The role of synaptic plasticity is to choose the optimal $\tau_{j}$ for each feature such that there will be a poststimulus timepoint, $t_{b}$, at which the frequencies become equal ($f_{b}$). These points are indicated by the green circles. Generally, plasticity acts to select long decay constants for early features and short ones for later features. (For interpretation of the references to colour in this figure legend, the reader is referred to the web version of this article.)

We are effectively assuming, following HB, that for each feature there are j=1..J neurons or neural circuits that respond initially at high frequency $f_{\max}$, and then with linearly reducing frequency specified by decay constants $\tau_{j}$. The role of synaptic plasticity is to choose the optimal $\tau_{j}$ for each feature so that there will be a poststimulus timepoint at which the frequencies become equal. This concept is described in Abbott (2001), and illustrated in Fig. 4. For example, if a word contains early onset of 1 kHz activity, then a long $\tau_{j}$ will be selected for that feature. If it contains late offset 1 kHz activity then a short $\tau_{j}$ will be selected for that feature.

In this paper we implement the plasticity process by simply computing those values of $\tau_{j}$ that will, given an initial frequency $f_{\max}$, make all the K frequencies equal to $f_{b}$ at time $t_{b}$. We refer to these optimal values as $\tau_{j_{\mathrm{opt}}}$. In the HB model it is spike timing that is synchronized and it is proposed that $\tau_{j_{\mathrm{opt}}}$ can be learnt via spike timing dependent plasticity (Lee, Sen, & Kopell, 2009). More recently, Gutig and Sompolinsky (2009) have shown that this can be implemented using a conductance-based tempotron. They also provide an analysis of the storage capacity of the HB-type coding scheme, estimating that it can store 0.0625 patterns per synapse. Thus, to store 5000 words would require 80,000 synapses.

#### 2.2.5 Time-warp invariance

**Fig. 5:**
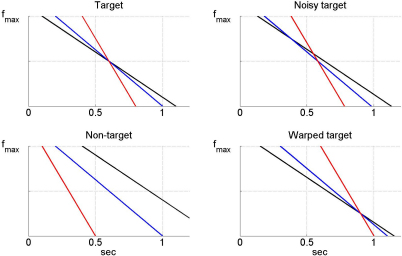
The top left figure shows the oscillator frequencies selected by the rightmost green circle in Fig. 4. The top right shows the same responses but for 5% noise added onto Occurrence Times. The bottom left shows the responses for a non-target pattern. Here, the ‘red’ feature now occurs before the ‘black’ feature so that there are no timepoints when the frequencies are similar. The bottom right figure shows responses for the target pattern but where Occurrence Times have been multiplied by 1.5. (For interpretation of the references to colour in this figure legend, the reader is referred to the web version of this article.)

Human speech is characterized by a four-fold variation in the speed at which words are spoken (Miller, Grosjean, & Lomanto, 1984), and any speech recognition system whether artificial or natural, will have to deal with this range of ‘time-warp’. In the above coding scheme time-warp invariance is achieved because the timing of the recognition event (gamma burst) depends on the speed at which the word is spoken. This is discussed at length in Hopfield and Brody (2001) and illustrated in Fig. 5. Time-warp invariance occurs rather naturally with OT features and WCO–TS/HB models but is more complicated to add to other representations. ASR based on cepstral coefficients, for example, requires an additional Dynamic Time Warping or Hidden Markov Modelling stage (Rabiner & Juang, 1993). What we have described so far is identical to the HB model. In fact, our dynamical pattern recognition model is the same as HB, except that the synchronization mechanism is instantiated in a WCO rather than an IF network, as described below.

#### 2.2.6 Weakly coupled oscillator network

Weakly coupled oscillators are a standard approach for studying synchronization dynamics (Hoppensteadt & Izhikevich, 1997). They can be derived by applying a phase reduction approach to neurophysiologically realistic neural network models. The only requirement is that the underlying neurons operate around a limit cycle and interact weakly (Brown et al., 2004; Ermentrout & Kleinfeld, 2001; Hansel et al., 1995). Cortical neuron models, for example, such as the Quadratic Integrate and Fire model or any model with ‘type 1’ dynamics (Hoppensteadt & Izhikevich, 1997), can be implemented as a ‘theta neuron’. This is a differential equation with a single phase variable, and a particular ‘phase interaction function’. Networks of such cells can then be analysed to find the neuronal mechanisms that give rise to synchronization. An early application, for example, used WCOs to infer that neuronal inhibition rather than excitation can cause synchronous activity (Vreeswijk et al., 1994).

More abstract models based on WCOs have also been used as neurocognitive models of visual attention (Corchs & Deco, 2001) and attention-guided object selection (Borisyuk & Kazanovich, 2004). These were based on previous models of visual attention (Niebur & Koch, 1994) and image segmentation (Wang & Terman, 1997) that also made use of synchronizing dynamics.

In the HB model it is the action potentials of IF neurons that become synchronized. Synchronization is brought about by balanced excitation and inhibition among excitatory and inhibitory IF cells. In this paper we are equivocal about the details of local neurons or neural circuits that bring about oscillation and synchronization (for reasons discussed above). Instead, we consider the properties of cells or circuits of cells using a description at the level of phase dynamics. Other than this difference, our model is more or less identical to that in HB.

It is assumed, as it is in the HB model, that Spike Timing Dependent Plasticity (STDP) is the underlying mechanism for forming ensembles of cells that synchronize together. Because cells must spike within a maximal period of 25 ms (approx) then the minimum frequency of the network oscillation is 40 Hz. Tighter synchronization will lead to higher frequency rhythms. This assumes that ECOG is detecting LFP activity of synchronized onset/offset detectors.

In a network of k=1 to K oscillators, each oscillates at unit amplitude with frequency $f_{k}\left(x,t\right)$ and phase $\phi_{k}\left(x,t\right)$ where x denotes the stimulus pattern and t denotes time. The rate of change of phase of the kth oscillator is given by

(3) $${\overset{˙}{\phi }}_{k}\left(x,t\right)=f_{k}\left(x,t\right)-\overset{K}{\underset{k^{\prime }=1}{\sum }}w_{kk^{\prime }}h\left[\phi_{k^{\prime }}\left(t\right)-\phi_{k}\left(t\right)\right]+e_{k}\left(t\right)$$

where $w_{kk^{\prime }}$ is the coupling strength between oscillators k and $k^{\prime },h\left[\Delta \phi \right]$ is a phase interaction function, and $e_{k}\left(t\right)$ is additive circular Gaussian noise and $f_{k}\left(x,t\right)$ is the frequency of the kth oscillator. In this paper the frequencies $f_{k}\left(x,t\right)$ are nonstationary and depend on the stimulus pattern x.

In this paper we make the simplifying assumption that the Phase Interaction Function (PIF) h(Δϕ)=sin(Δϕ) which results in the Global Zero Lag (GZL) solution (where all phase differences are zero—i.e., full synchronization), being a potential stable state of the system (Ermentrout & Kleinfeld, 2001; Penny et al., 2009). We envisage that in future work it might be possible to infer h based on neuroimaging data, an approach we have implemented for magnetoencephalograph data (Penny et al., 2009).

The noise is drawn from a circular Gaussian (von-Mises) density with zero mean and precision κ. This particular form was chosen as it is the simplest density that gives rise to noise that is bounded between 0 and 2π. The inclusion of noise was found to provide better fits to the ECOG spectrograms (see Section 3.2). For the results below we used a value of κ=10 and used ‘frozen noise’ by drawing $e_{k}\left(t\right)$ prior to each model fitting run.

WCO models are often studied with the assumption that the frequencies are stationary, $f_{k}\left(x,t\right)=f_{k}\left(x\right)$. Much research examines the robustness of stable synchronized states as a function of variations in oscillator frequencies. Kuramoto (1984), for example, has derived the following result. If the frequencies are Lorentz distributed with variance $\sigma_{L}^{2}$, then a stable synchronized state will be reached if the coupling parameters $w_{kk^{\prime }}=w$ and satisfy

(4) $$\frac{\sigma_{L}}{wK}<\frac{1}{2}\text{.}$$

This shows that if the frequencies are more heterogeneous then stronger coupling is needed to reach a synchronized state. It is not specified, however, how long it takes for synchronization to be achieved so this result is not of immediate relevance to the WCO–TS model.

In previous work we have used WCOs to study synchronization among different brain regions (Penny et al., 2009) whereas in this paper we use them to model activity in a single region. Specifically, the Local Field Potential (LFP) in a region is modelled as

(5) $$y\left(x,t\right)=\overset{K}{\underset{k=1}{\sum }}\cos\left[\phi_{k}\left(x,t\right)\right]\text{.}$$

If the oscillators are phase aligned then the field activity will reach a maximum value K. Weaker synchronization results in a smaller LFP. This is shown in the top row of Fig. 3 where we have a large gamma burst in response to a word and a small one in response to a nonword (a pattern which the network has not previously been exposed to).

A quantitative relationship can be derived, for example, by assuming that the instantaneous phases are Gaussian distributed with phase variance $\sigma_{t}^{2}$. The instantaneous field power is then given by (Roweis, 2009)

(6) $$\langle y{\left(x,t\right)}^{2}\rangle =1+e^{-\sigma_{t}^{2}}\left(K-1\right)\text{.}$$

This completes the description of the WCO network.

### 2.3 Modelling electrocorticogram data

For modelling the ECOG data, the input frequency to the kth oscillator in the WCO network is given by

(7) $$f_{k}\left(x,t\right)=f_{kj_{\mathrm{opt}}}\left(x,t\right)+g_{k}$$

where $f_{kj_{\mathrm{opt}}}\left(x,t\right)$ is defined in Eq. (2) with $j=j_{\mathrm{opt}}$, and $g_{k}$ the baseline frequency of the kth oscillator. This baseline determines the oscillation frequency before and after the stimulus-induced transient. We define an average baseline frequency for the ensemble, g, and then draw $g_{k}$ from a uniform distribution centred on g and with a bandwidth of g/2. In this paper we use g=10Hz so as to reflect typical background alpha activity (Buzsaki, 2006).

Critically, if the input pattern x is a nonword then we assume that the $\tau_{j}$’s will not have been optimized for the features $t_{k}\left(x\right)$. For nonwords we use the $\tau_{j}$’s associated with the corresponding paired word. For nonwords the input frequencies $f_{k}\left(x,t\right)$ will then not be all equal at time $t_{b}$ and there will be no LFP gamma burst (or it will be greatly reduced in power). This is illustrated in the top two rows of Fig. 3.

To fit the ECOG spectrogram we wish to obtain the difference in spectral responses between words and nonwords. This could be implemented by computing the field activity for all C oscillators in the pattern recognition region. However, in this paper we make a computational saving by considering only the field contribution from those cells that are activated by both stimulus patterns of a given word/nonword pair (note that for a given input not all cells may be activated as the onset/offset/peak response may occur after the cut-off time of $t_{k}\leq 300\;\mathrm{ms}$—see above)

In early experiments with the WCO–TS model we became concerned that bursts of activity would also emerge at resting frequencies. We therefore considered an augmented model in which coupling parameters were allowed to be frequency dependent, as described in the Appendix B (but see results).

#### 2.3.1 Spectrograms

We choose i=1 to N pairs of word exemplars, $x_{wi}$, and nonword exemplars, $x_{ni}$. For each pair we compute the local field responses $y\left(x_{wi},t\right)$ and $y\left(x_{ni},t\right)$ by first integrating the WCO dynamics (Eq. (3)) using the Euler–Maruyama method (Kloeden & Platen, 1999) to compute the phase time series for the network of oscillators, $\phi_{k}$, and then use Eq. (5) to produce the field response. Examples of these LFPs are shown in the top row of Fig. 3.

We also considered an augmented model in which burst time and frequency ($t_{b}$ and $f_{b}$) were allowed to vary over trials. We considered variations of the form

(8) $$t_{b}^{i}=\left(1-\delta t\right)t_{b}+2\delta tz_{i}$$

$$f_{b}^{i}=\left(1-\delta f\right)f_{b}+2\delta fw_{i}$$

where δt and δf are parameters to be estimated, and $\left\{z_{i},w_{i}\right\}$ are random variables uniformly distributed between 0 and 1.

The corresponding spectrograms $G\left(x_{wi},f,t\right)$ and $G\left(x_{ni},f,t\right)$ are computed using the windowed multi-taper method described above, using identical parameters as for the ECOG data itself. It is then possible to compute the average spectral difference between word and nonword responses

(9) $$D_{s}\left(f,t;\theta \right)=\frac{1}{N}\overset{N}{\underset{i=1}{\sum }}\left[G\left(x_{wi},f,t\right)-G\left(x_{ni},f,t\right)\right]$$

where θ are model parameters (see below). Upon analysing the auditory data files we noticed a systematic bias in mean OTs between words and nonwords (123 ms for nonwords versus 140 ms for words, p=0.04). As we did not wish this to unduly influence the synchronization processes we adopted the following procedure. We first define the spectral difference that would be obtained without any synchronization (this is obtained by setting the coupling A=0)

(10) $$D_{0}\left(f,t;\theta \right)=\frac{1}{N}\overset{N}{\underset{i=1}{\sum }}\left[G_{0}\left(x_{wi},f,t\right)-G_{0}\left(x_{ni},f,t\right)\right]\text{.}$$

The predicted spectral difference from the WCO–TS model is then given by

(11) $$D\left(f,t;\theta \right)=D_{s}\left(f,t;\theta \right)-D_{0}\left(f,t;\theta \right)\text{.}$$

#### 2.3.2 Parameter estimation

Our model contains stochastic variables such as the state noise, $e_{k}\left(t\right)$, and for the augmented models, the between trial variables $\left\{z_{i},w_{i}\right\}$. These random variables were drawn prior to each model fitting run. The use of such ‘frozen noise’ ensures that models with the same parameters have the same likelihood, a requirement of the model fitting procedure.

The other parameters, θ, (see Tables 1 and 2) were estimated from data. This data comprise a subset of the (paired) word and nonword utterances (auditory inputs and ECOG spectrograms). The overall set of 91 exemplars was split into a training set of 42 exemplars, used to estimate model parameters, and a test set of 51 exemplars (the other 3 outlying exemplars were removed). Model fitting was implemented using a Bayesian parameter estimation algorithm as follows.

To obtain a quantitative measure of how well the data and model spectrograms are matched we first normalize both to have unit power over the specified time interval. We then project D(f,t) onto the first eigenvector of Y(f,t). Model error, E(θ), is then computed as the Root Mean Squared (RMS) difference between the resulting projections. Log model likelihood is defined to be proportional to negative model error

(12) p(Y|θ)∝exp[−E(θ)].

We placed uniform priors over model parameters as shown in Tables 1 and 2. The posterior density was then estimated using a Metropolis–Hastings (MH) algorithm (Gelman, Carlin, Stern, & Rubin, 1995). This uses a proposal density which we chose to be a zero-mean diagonal covariance Gaussian with standard deviation for the pth parameter given by

(13) $$\sigma_{p}=\frac{\theta_{\max}^{p}-\theta_{\min}^{p}}{S}$$

where $\theta_{\min}^{p},\theta_{\max}^{p}$ are defined in Tables 1 and 2, and S was chosen to achieve high acceptance rates (see results). At each iteration of the MH algorithm a proposal, $\theta^{*}$, is generated by adding a sample from the proposal density onto the sample from the previous iteration $\theta^{n-1}$. Proposals that fell outside the range of the uniform prior were immediately rejected. Other proposals were accepted with probability

(14) $$\min\left(\frac{p\left(\theta^{*}|Y\right)}{p\left(\theta^{n-1}|Y\right)},1\right)\text{.}$$

If the proposal is accepted we set $\theta_{n}=\theta^{*}$, and if not then $\theta_{n}=\theta_{n-1}$. The above MH criterion ensures that, after a burn in period, the algorithm produces samples from the posterior density of interest (Gelman et al., 1995). This posterior distribution (as with any Bayesian estimation) takes into account both the uniform priors over model parameters (see Tables 1 and 2) and the fit of the model to the data.

## 3 Results

The first results section examines the suitability of OT features for speech recognition, by using the multiple speaker isolated word database described in Hopfield and Brody (2000, 2001). The second results section examines the use of the overall dynamic pattern recognition process as a forward model of Electrocorticogram data.

### 3.1 Pattern recognition

We now demonstrate the usefulness of Occurrence Times (OTs) as speech recognition features by comparing them to a more commonly used feature, Mel-Frequency Cepstral Coefficients (MFCCs) (Roweis, 1998). MFCCs form the front-end of state-of- the-art speech recognition systems such as HTK (Woodland, 2003) and Sphinx-4 (Walker, Lamere, Kwok, Raj, & Singh, 2004). To simplify the comparison of MFCC and OT features they were both fed into an identical pattern recognition stage, chosen to be a first nearest neighbour classifier (Duda & Hart, 1973).

We used the multiple speaker isolated word database described in Hopfield and Brody (2000, 2001). This comprises 500 speech files, the words ‘zero’, ‘one’, through to ‘nine’, spoken by five different female speakers with ten replications of each word per speaker. This data is a subset of the TI46 database from the Linguistic Data Consortium (Verstraeten, Schrauwen, Stroobandt, & Campenhout, 2005). The data was partitioned into a fixed training set, comprising 5 utterances per digit per speaker, and a fixed test set holding the remaining 5 utterances per digit per speaker. This gives 250 training and 250 testing exemplars. We first report recognition results on this noiseless isolated word data set and then go on to test the systems in the presence of additive noise. The Word Error Rates (WERs) reported below refer to the error rate on the test set.

The MFCCs were computed using a standard processing pipeline as described in the Appendix A. One issue here arises from the fact that the speech time series must first be partitioned into frames, and the overall MFCC vector is then concatenated over frames. As the speech signals were, however, of different lengths and comparison of feature vectors (see below) requires them to be of the same length we derived features for a fixed time period $t_{\mathrm{fix}}\;\mathrm{ms}$. Blocks for which signals were unavailable (e.g., due to words being shorter than $t_{\mathrm{fix}}\;\mathrm{ms}$) were assigned MFCC values of zero. This coding also provides discriminatory information (e.g., that word A is shorter than word B). We also tried setting $t_{\mathrm{fix}}$ to the length of the shortest word in the database, but this resulted in much worse classification performance. We varied various parameters of the MFCC processing to achieve optimal performance. The best performance, a Word Error Rate (WER) of 0.000 (perfect discrimination), was achieved with K=18 cepstral coefficients per frame. Other parameters were set as described in the Appendix A.

The fact that different speech signals were of different length provided no problem for OT features as a fixed length code was always readily obtainable (each channel always has an onset, peak and offset regardless of the amount of data). The onsets, peaks and offsets were defined as described earlier. We also implemented robustness to global time-warps by dividing all OT values by the time between the first and last OT.

We used a fixed number B=11 frequency bins. This system produced WER=0.100. We then improved the system by introducing multiple level detectors for each onset and offset feature, as proposed in Gutig and Sompolinsky (2009). This additional ‘intensity encoding’ reached an optimal level of performance with 7 intensity levels per detector, with WER=0.024. Thus, for each of the 11 frequency bands there is one maximum detector, 7 level crossings for onsets and 7 level crossings for offsets. Overall, that is 15 features per frequency band giving a total of 165 features. In the original HB paper it was proposed that performance might be further improved if multiple crossings of each intensity level were allowed. We implemented this but did not find a reduction in WER on this database.

**Fig. 6:**
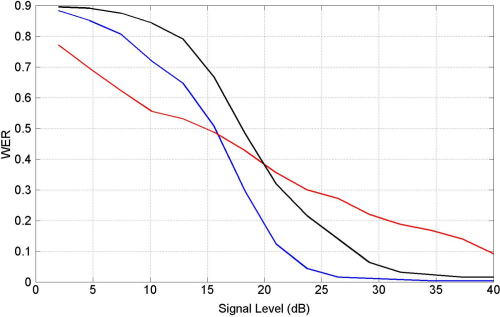
Speech recognition in additive white noise. We plot Word Error Rate (WER) against signal level for optimized speech recognition systems using Occurrence Time (OT) features (red curve), Mel Frequency Cepstral Coefficients (MFCC) (blue curve) and MFCC coefficients but with the number of features matched to that of the OT system (black curve). (For interpretation of the references to colour in this figure legend, the reader is referred to the web version of this article.)

Thus, on the noiseless data we can conclude that OT features with multiple levels of intensity encoding provide reasonable recognition performance, though not as good as MFCCs. We now take the optimized MFCC and OT systems and apply them to noisy data. We first corrupted the test data with white noise to produce a range of signal to noise ratios. We define the signal level, measured in decibels (dB), as $S=20\log_{10}\left(\sigma_{s}/\sigma_{e}\right)$ where $\sigma_{s}$ is the signal standard deviation and $\sigma_{e}$ is the noise standard deviation. Fig. 6 shows that speech recognition performance rapidly degrades at less than 25 dB, as is well known (Ghitza, 1986). It also shows that the MFCC system is better for high signal levels whereas the OT system is better for low signal levels.

We thought it might be possible that the OT system performed better at low signal levels because it had fewer parameters than the MFCC system, and so might generalize better (Bishop, 1995). We therefore applied an MFCC system that was matched in the number of parameters, but this did not improve performance at low signal levels (see black curve in Fig. 6).

**Fig. 7:**
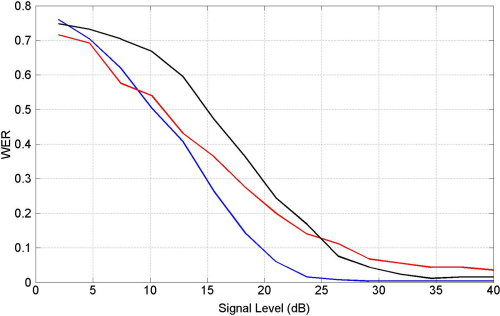
Speech recognition in additive speech babble. We plot Word Error Rate (WER) against signal level for optimized speech recognition systems using Occurrence Time (OT) features (red curve), Mel Frequency Cepstral Coefficients (MFCC) (blue curve) and MFCC coefficients but with the number of features matched to that of the OT system (black curve). (For interpretation of the references to colour in this figure legend, the reader is referred to the web version of this article.)

Finally, we repeated the speech recognition tests with additive speech ‘babble’ which was derived from 100 people speaking in a canteen (this data was downloaded from the Signal Processing Information Base http://spib.rice.edu). The results in Fig. 7 show that MFCC is better for high signal levels but that OT and MFCC have the same performance at low signal levels.

We conclude that OT features provide a compact code for auditory word recognition that rivals that of standard encoding methods in noisy environments. An important caveat here is that we have used the same back-end for both OT and MFCC features, namely a nearest neighbour classifier. This back-end is not optimal for either front-ends, but serves to provide a common baseline for both approaches. MFCC features are much more powerful when used in combination with an HMM classifier (Woodland, 2003), and OT features when used with a recognition process such as a Tempotron (Gutig & Sompolinsky, 2009). We return to this issue in the discussion.

### 3.2 Modelling electrocorticogram data

This section describes the use of the WCO–TS network as a forward model of ECOG data. The networks are driven by auditory data (word versus nonword). As described in Section 2.1, this data set comprises i=1..96 word utterances and i=1..96 paired nonword utterances. For each utterance we have the original auditory data file ($y_{wi}$ and $y_{ni}$ for words and nonwords) and a spectrogram of the corresponding ECOG response, $G\left(y_{wi},f,t\right)$ and $G\left(y_{ni},f,t\right)$.

For each utterance, the auditory signal, x(t), produces bandpass filtered responses (described in Section 2.2.1), which in turn produce Occurrence Time features (Section 2.2.2) in a bank of feature detectors. Each detector oscillates initially at some maximum frequency which then decreases due to spike rate adaptation. Oscillator synchronization then produces a field potential from the WCO–TS network as described in the remainder of Section 2.2. The data set of (paired) word and nonword utterances (96 exemplars, comprising auditory inputs and ECOG spectrograms) are then split into a training set of 42 exemplars, and a test set of 51 exemplars (3 outlying exemplars were removed).

The Bayesian parameter estimation algorithm (Section 2.3.2) was run on data from the 42 training exemplars. For the proposal densities in the Metropolis–Hastings algorithm we used a value of S=8 as this achieved a high acceptance rate. We ran the algorithm for 2000 iterations and discarded samples from the first 1000 iterations. For each sample, approximately 15 s of computer time were required to compute the likelihood (using a 64-bit dual core IBM with 12 G RAM and 3.2 GHz clock speed). Overall, model fitting required about 8 h of computer time. Model fits were assessed using a likelihood function that is related to the RMS error between training data and model spectrograms (see Eq. (12)).

A numerical benefit of describing transient synchronization by phase dynamics (Eq. (3)), rather than more detailed IF network models (Hopfield & Brody, 2001), is that they describe a similar phenomenon but are quicker to numerically integrate. For the WCO integration we used a step size of 2.5 ms (the limit on the step size is due to the sampling rate we need to estimate the frequencies produced).

**Fig. 8:**
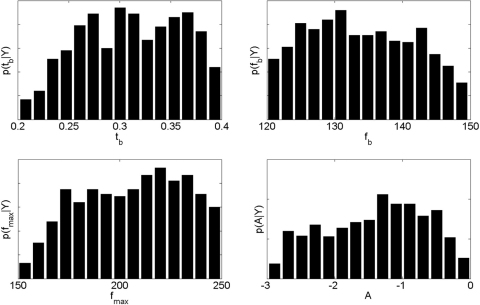
Posterior densities over burst time $t_{b}$, burst frequency $f_{b}$, coupling parameter A, and maximum frequency $f_{\max}$. The values in the histograms sum to unity as they depict probability densities.

**Fig. 9:**
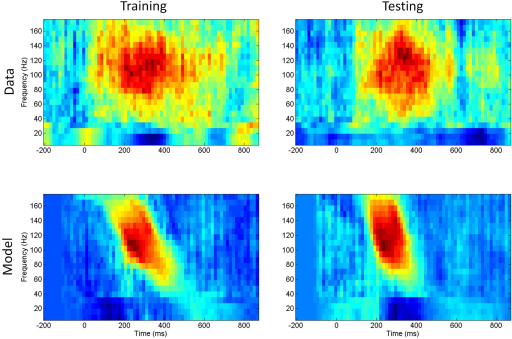
*Minimal model* Top row: data spectrogram for training set (left) and test set (right). Bottom row: model spectrogram for training set (left) and test set (right) computed using a high-likelihood sample from the posterior density.

We first present results from the minimal model (model M0) which used just four adaptable parameters ($t_{b},f_{b},f_{\max},A$). Fig. 8 shows posterior distributions of these parameters as estimated using the MH algorithm. The fairly flat distributions indicate that data fit is relatively insensitive to the exact values of these parameters. This is a sign of a good model. Fig. 9 (left column) shows the data and model spectrograms for the maximum posterior sample.

The performance of the model was then evaluated on an independent test set comprising 51 exemplars (see above). Data and model spectrograms for the test examples are shown in Fig. 9 (right column). Both training and test data show good agreement between data and model spectrograms.

**Fig. 10:**
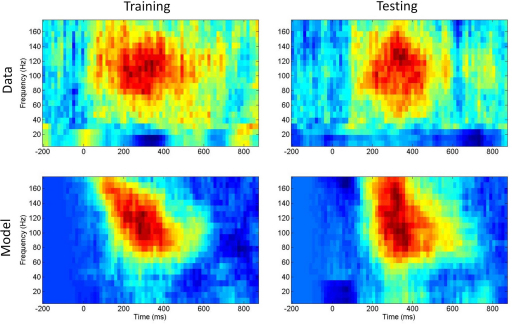
*Augmented model* Top row: data spectrogram for training set (left) and test set (right). Bottom row: model spectrogram for training set (left) and test set (right) computed using a high-likelihood sample from the posterior density.

We now present the results of an augmented model (model M2) which also contained frequency dependent synchronization and between-trial burst time/frequency variability (see Table 2). Fig. 10 shows the data and model spectrograms.

We also compare augmented and minimal models using the model evidence, as computed using the Posterior Harmonic Mean (PHM) (Gelman et al., 1995). This approximates the evidence for a model using samples from the posterior density

(15) $$p_{\mathrm{PHM}}\left(Y|M\right)={\left[\frac{1}{N_{s}}\overset{N_{s}}{\underset{n=1}{\sum }}\frac{1}{p\left(Y|\theta_{n},M\right)}\right]}^{-1}$$

where $p\left(Y|\theta_{n},M\right)$ is the likelihood of the nth posterior sample, and $N_{s}=1000$. We compared the minimal model (M0) to two augmented models, one with between-trial burst time/frequency variability (M1), and one with both frequency dependent synchronization and between-trial burst time/frequency variability (M2). The resulting Bayes factors were 0.98 and 0.99 indicating that neither of the augmented models are significantly better than the minimal model (this would require a Bayes factor of at least three (Gelman et al., 1995)).

## 4 Discussion

This paper has described a dynamical process which serves both as a model of temporal pattern recognition in the brain and as a forward model of neuroimaging data. This work is based heavily on prior developments by Hopfield and Brody (2001) and we have made three novel contributions to the literature.

First, we have viewed the HB model at two separate levels of analysis; the algorithmic and implementation levels (Marr & Poggio, 1976). Algorithmically, the HB model is marked out by its use of Occurrence Times (OTs) as features. We have shown using a nearest neighbour classifier that, for noisy recognition environments, OT features rival standard MFCC features in classification accuracy. For non-noisy data MFCC features were found to be better.

An important caveat to the above finding is that we used the same back-end for both OT and MFCC features, namely a nearest neighbour classifier. Moreover, this back-end is not optimal for either front-ends, but serves to provide a common baseline for both approaches. MFCC features are much more powerful when used in combination with an HMM classifier (Woodland, 2003).

For example, an MFCC–HMM system can achieve a word error rate of only 11% for connected digits in noise, with an additive noise level of 10 dB (Lee, Glass, & Ghitza, 2011). A similar level of performance is obtained when using ecologically realistic noise samples from the Aurora-2 database (Pearce & Hirsch, 2000). This is to be contrasted with the relatively poor performance of the MFCC–NN system obtained in this paper on the simpler recognition problem of isolated digits in noise (see Figs. 6 and 7), where we obtain a word error rate of about 70%. We should also bear in mind, however, that our results were obtained by training the system on clean utterances whereas the results in Lee et al. (2011) were obtained from a system trained on noisy utterances. Our results are more in line with those of Rouat, Loiselle, and Molotchnikoff (2011) who obtained word error rates of 78% when training an MFCC–HMM system on clean utterances and testing it on 10 dB noisy utterances using noise samples from Aurora-2.

We also note that OT features are also more powerful when used with a matched, optimized recognition process such as a Tempotron. An OT-Tempotron (Gutig & Sompolinsky, 2009) matched the performance level of MFCC–HMM approaches implemented in the HTK (Woodland, 2003) and Sphinx 4 (Walker et al., 2004) ASR systems, on noiseless isolated word recognition.

Second, we have proposed a generic model of transient synchronization based on Weakly Coupled Oscillators. This has allowed us to focus on the dynamics of transient synchronization per se rather than on the neural mechanisms by which the underlying oscillations are generated. As the way in which one cell or circuit couples with another can be summarized using ‘phase interaction functions’ (Penny et al., 2009) we envisage that it should be possible to identify families of neurons or neural circuits that have the appropriate synchronization properties.

Third, we have shown that the dynamical pattern recognition process can act as a forward model of neuroimaging data. Previous studies in this area (Corchs & Deco, 2004; Husain, Tagamets, Fromm, Braun, & Horwitz, 2004) have used computational models of auditory processing as forward models for fMRI data. Corchs and Deco (2004), for example, have used a neurodynamical model of feature-based attention in combination with a haemodynamic process as a forward model of fMRI activity. Husain et al. (2004) have taken a similar approach using a large-scale neural network of the auditory system as a model of fMRI activity. We have used ECOG data which, having a higher temporal resolution than fMRI data, has allowed us to focus on the dynamics on the recognition process itself. We were also able to show how the parameters of our model can be directly fitted to neuroimaging data using Bayesian inference. This follows the example set by Dynamic Causal Modelling (Friston et al., 2003). The empirical work in this paper shows that a minimal model using only four parameters is able to provide a good fit to our particular ECOG data set.

In the HB model, synchronization of spike timing is achieved through balanced excitation and inhibition of ensembles of integrate and fire cells. This is a perfectly plausible mechanism and may indeed be an accurate description of how cells in the brain synchronize. As we have described in the introduction there are multiple alternative ways that cells might synchronize. The WCO-based recognition-by-synchronization module might be seen as an improvement on the HB module in two ways. First, it is less committed to a specific biophysical synchronization mechanism, which we see to be an advantage because the mechanism by which neurons in the mammalian auditory synchronize is currently unknown. Second, when used as a forward model of neuroimaging data it is computationally more efficient because a larger integration step size can be used.

The TS mechanism we have investigated is similar to other models of neural processing that rely on transient dynamics (Rabinovich, Huerta, & Laurent, 2008). The Liquid State Machine (LSM) (Maass, Natschlager, & Markram, 2002), for example, uses OT features and the temporal embedding idea proposed in the HB model, but then applies standard methods for recognizing the resulting static patterns. This results in good pattern discrimination abilities (Verstraeten et al., 2005), though not as accurate as a recent approach based on OT features (Gutig & Sompolinsky, 2009). Further, LSMs do not generate a gamma burst as an integral part of the recognition process, so would not be so appropriate as a forward model for the sort of neuroimaging data addressed here.

The notion that regions higher up in the auditory cortical hierarchy process information at longer time scales has recently been made use of in a model of auditory sequence recognition based on stable heteroclinic channels (Kiebel, Kriegstein, Daunizeau, & Friston, 2009). Moreover, the approach developed in that work derives from a Bayesian perspective in which cortical hierarchies embody a generative model which is then inverted during the pattern recognition process. Generative models of speech production are, as yet however, still in the early stages of development. This currently limits the ecological validity of such a generative modelling approach.

The importance of a hierarchy of temporal scales is emphasized in recent work by Ghitza (2011) who provides evidence that current models of speech perception, which are driven by acoustic features alone, provide an incomplete description of speech recognition phenomena. An alternative description which highlights the role of decoding time provides a better match to human perceptual performance, and they suggest that decoding time is governed by a cascade of neural oscillators operating at different time scales.

Finally, we have used the dynamic pattern recognition model to predict activity in only a single brain region, the posterior superior temporal sulcus. This region has been identified in several fMRI paradigms where normal speech is compared with various unintelligible speech foils (Leff et al., 2008; Obleser, Wise, Dresner, & Scott, 2007; Vouloumanos, Kiehl, Werker, & Liddle, 2001). Given that task-dependent BOLD responses and gamma oscillations are coupled, it seems reasonable to suggest that the activations reported in these studies could be driven by the gamma burst predicted to occur when spike time synchronization occurs. This response will be more robust for sensory items with long-term neural representations maintained by repeated exposure (i.e., words), compared with acoustically comparable items that do not (i.e., non-words).

Whilst the work in this paper provides a useful starting point, it does not make use of the network view of brain function; Price, Thierry, and Griffiths (2005), for example, propose that the human brain has not developed macro-anatomical structures dedicated to speech processing, but rather that speech-specific processing emerges at the level of functional connectivity among distributed regions. The ECoG data we have analysed has recordings of activity from 64 electrodes placed over fronto-temporal cortex, yet we have modelled data from only a single electrode. Extension of our modelling to include multiple regions, as in Corchs and Deco (2004); Husain et al. (2004), is therefore an important direction for future work.

## Figures and Tables

Spectral Analysis of ECOG data from Canolty et al. (2007).

**Table 1:** *Minimal model* uniform priors over model parameters. The minimal model used four parameters only: burst time ($t_{b}$), burst frequency ($f_{b}$), maximum frequency ($f_{\max}$), and coupling strength (A).

Parameter	Minimum	Maximum
$t_{b}$ (ms)	200	300
$f_{b}$ (Hz)	120	150
$f_{\max}$ (Hz)	150	250
A	−3	0

**Table 2:** *Augmented model* uniform priors over model parameters. Augmented models used the same parameters as for the minimal model but additionally had parameters for (i) frequency dependent synchronization ($\beta_{1},\beta_{2}$) and/or (ii) between-trial burst time/frequency variability (δt,δf). The $\delta_{t}$ and $\delta_{f}$ parameters, for example, allow for between a 10% and 30% trial to trial variability in burst time/frequency.

Parameter	Minimum	Maximum
$\beta_{1}$	−0.25	0.25
$\beta_{2}$	0.6	0.9
δt	0.1	0.3
δf	0.1	0.3

## Appendix A Mel frequency cepstral coefficients

The MFCCs were derived using a standard data processing pipeline (Gutig & Sompolinsky, 2009). As the magnitude spectrum of speech is known to be stationary over a period of approximately $t_{\mathrm{win}}=20\text{–}100\;\mathrm{ms}$ (Rabiner & Juang, 1993) we broke up each speech time series into frames of length $t_{\mathrm{win}}=50\;\mathrm{ms}$. These frames were offset from each other by $t_{\mathrm{off}}=50\;\mathrm{ms}$, resulting in non-overlapping frames. As the overall time series was $t_{\mathrm{fix}}=900\;\mathrm{ms}$, this resulted in 18 frames. The frame size and offset are a little larger than is standard but these settings were found to give perfect speech recognition performance on non-noisy data (see main text).

For each frame of data we first applied a pre-emphasis filter

(16) $${\overset{ˆ}{x}}_{n}=x_{n}-\alpha x_{n-1}$$

with α=0.97. We then applied a 512 point FFT to the time series ${\overset{ˆ}{x}}_{n}$ to obtain the power spectrum $p_{x}$. This was then multiplied by a set of k=1..K filters (see below), $s_{k}$, to give

(17) $$z_{k}=s_{k}p_{x}\text{.}$$

The MFCCs were then computed as

(18) a=DCT(log(z))

where DCT is the Discrete Cosine Transform. Thus, the MFCCs are given by a cosine transform of the logarithm of the filtered power spectra. The filters are triangular-shaped and spaced uniformly on the ‘mel-frequency scale’

(19) $$f_{m}=2595\log_{10}\left(1+f/700\right)$$

where f is the frequency in Hz and $f_{m}$ is the frequency in ‘mels’. The mel scale has been designed such that intervals are equally separated in perceptual distance, with $f_{m}=1000$ mels being equivalent to f=1000Hz. The relationship is linear below 1000 Hz and sub-linear above it.

The cepstral parameters were then weighted by multiplying the kth parameter, $a_{k}$, by

(20) $$w\left(k\right)=1+\frac{K}{2}\sin\left(\frac{k}{K}\pi \right)$$

for k=1..K, a procedure known as ‘liftering’. The first and second order derivatives of the MFCCs (with respect to time) are sometimes used as additional features (Gutig & Sompolinsky, 2009) but did not improve recognition performance on our database.

## Appendix B Frequency dependent coupling

The main text describes a WCO–TS model based on uniform coupling, $w_{kk^{\prime }}=A$. We also allowed for an augmented model in which coupling strength also depends on the mean frequency of the ensemble,

(21) $$\overset{¯}{f}\left(x,t\right)=\frac{1}{K}\overset{K}{\underset{k=1}{\sum }}f_{k}\left(x,t\right)\text{.}$$

This takes the sigmoidal form

(22) $$w_{kk^{\prime }}=\frac{A}{1+\exp\left(-a\right)}$$

$$a=\beta_{1}\left(\overset{¯}{f}\left(x,t\right)-\beta_{2}f_{b}\right)\text{.}$$

 The purpose of this frequency-dependence is to reduce synchronization at lower frequencies. The possibility that synchronization increases with frequency is commensurate with in-vitro cell recordings (Rocha, Doiron, Shea-Brown, Josic, & Reyes, 2007) and computer simulation of both integrate and fire and Hodgkin–Huxley type models (Chawla, Lumer, & Friston, 1999).
